# 5,11,17,23,29,35-Hexa-*tert*-butyl-37,38,39,40,41,42-hexa­kis­(eth­oxy­carbonyl­meth­oxy)calix[6]arene acetonitrile disolvate

**DOI:** 10.1107/S1600536812015735

**Published:** 2012-04-18

**Authors:** Michaela Pojarová, Michal Dušek, Jan Budka, Ivana Císařová, Emanuel Makrlík

**Affiliations:** a Institute of Physics, AS CR, v.v.i., Na Slovance 2, 182 21 Praha 8, Czech Republic; b Institute of Chemical Technology, Technická 5, 166 28 Prague 6, Czech Republic; c Department of Inorganic Chemistry, Charles University in Prague, Faculty of Natural Sciences, Hlavova 2030/8, Praha, Czech Republic, 128 40, Czech Republic; d Faculty of Environmental Sciences, Czech University of Life Sciences, Prague, Kamýcká 129, 165 21 Prague 6, Czech Republic

## Abstract

In the title compound, C_90_H_120_O_18_·2CH_3_CN, the calix[6]arene has a 1,2,3-alternate conformation and possesses inversion symmetry. It crystallizes as an acetonitrile disolvate, with a half-mol­ecule of calix[6]arene and one mol­ecule of solvent in the asymmetric unit. In the crystal, the two solvent mol­ecules are enclosed in voids between the calix[6]arene mol­ecules. They form weak C—H⋯O hydrogen bonds involving an O atom of the lower rim substituent. The cavity of the calix[6]arene itself is enclosed by two opposite phenol rings, which are turned into the cavity due to the presence of a C—H⋯π inter­action. The calix[6]arene mol­ecule exhibits disorder of one substituent on its lower rim [occupancy ratio 0.897 (3):0.103 (3)].

## Related literature

For general information about calixarenes, see: Gutsche (2008 ▶). For their applications in coordination chemistry, see: Homden & Redshaw (2008 ▶); Gibson *et al.* (1998 ▶), in supra­molecular chemistry, see: Atwood *et al.* (2002 ▶) and in polymerization, see: Ling *et al.* (2003 ▶). For the synthesis of the title compound, see: McKervey *et al.* (1985 ▶). 
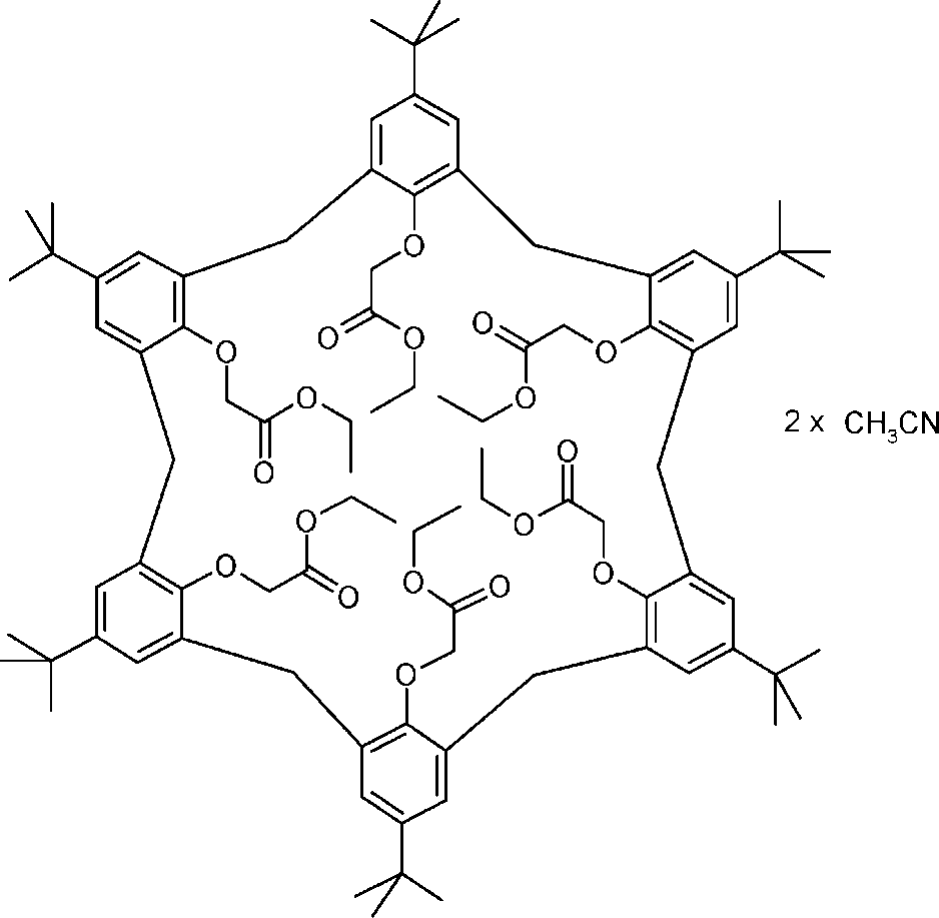


## Experimental

### 

#### Crystal data

C_90_H_120_O_18_·2C_2_H_3_N
*M* *_r_* = 1571.97
Triclinic, 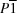

*a* = 12.6190 (2) Å
*b* = 13.1500 (3) Å
*c* = 14.8990 (4) Å
α = 75.9037 (11)°
β = 67.7646 (11)°
γ = 74.5815 (19)°
*V* = 2177.67 (9) Å^3^
*Z* = 1
Mo *K*α radiation
μ = 0.08 mm^−1^
*T* = 150 K
0.3 × 0.3 × 0.25 mm

#### Data collection

Nonius KappaCCD area-detector diffractometer
19018 measured reflections
9990 independent reflections
7277 reflections with *I* > 2σ(*I*)
*R*_int_ = 0.025

#### Refinement

*R*[*F* ^2^ > 2σ(*F* ^2^)] = 0.061
*wR*(*F* ^2^) = 0.183
*S* = 1.03
9990 reflections
538 parameters
12 restraints
H-atom parameters constrained
Δρ_max_ = 0.43 e Å^−3^
Δρ_min_ = −0.32 e Å^−3^

### 

Data collection: *COLLECT* (Hooft, 1998 ▶); cell refinement: *COLLECT*; data reduction: *COLLECT* and *DENZO* (Otwinowski & Minor, 1997 ▶); program(s) used to solve structure: *SHELXS97* (Sheldrick, 2008 ▶); program(s) used to refine structure: *SHELXL97* (Sheldrick, 2008 ▶); molecular graphics: *Mercury* (Macrae *et al.*, 2006 ▶) and *ORTEP-3* (Farrugia, 1997 ▶); software used to prepare material for publication: *publCIF* (Westrip, 2010 ▶).

## Supplementary Material

Crystal structure: contains datablock(s) I, global. DOI: 10.1107/S1600536812015735/su2398sup1.cif

Structure factors: contains datablock(s) I. DOI: 10.1107/S1600536812015735/su2398Isup2.hkl

Additional supplementary materials:  crystallographic information; 3D view; checkCIF report

## Figures and Tables

**Table 1:** Hydrogen-bond geometry (Å, °) *Cg*1 is the centroid of the C1*C*–C6*C* ring.

*D*—H⋯*A*	*D*—H	H⋯*A*	*D*⋯*A*	*D*—H⋯*A*
C3—H3*E*⋯O1*C*	0.96	2.47	3.250 (4)	139
C7*C*—H7*C*2⋯O3*B*	0.97	2.38	3.312 (3)	160
C12*B*—H12*D*⋯O2*A*	0.97	2.57	3.526 (3)	169
C15*B*—H15*G*⋯O3*A*^i^	0.96	2.48	3.398	160
C5*C*—H5*C*⋯O1*A*^ii^	0.93	2.44	3.190 (2)	138
C11*C*—H11*G*⋯O1*B*^ii^	0.96	2.56	3.477 (3)	159
C10*C*—H10*I*⋯*Cg*1^ii^	0.96	2.73	3.588 (3)	148

Symmetry codes: (i) 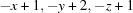; (ii) 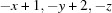.

**Fig. 1.**
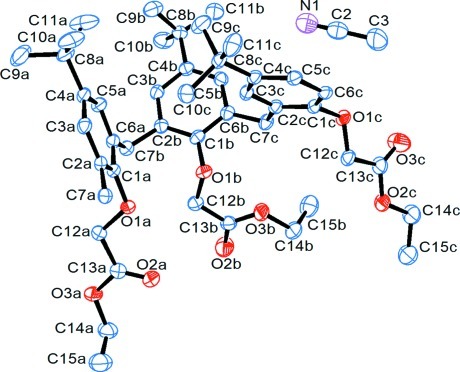
A view of the asymmetric unit of the title compound with atom numbering. Displacement ellipsoids are shown at the 50% probability level.

**Fig. 2.**
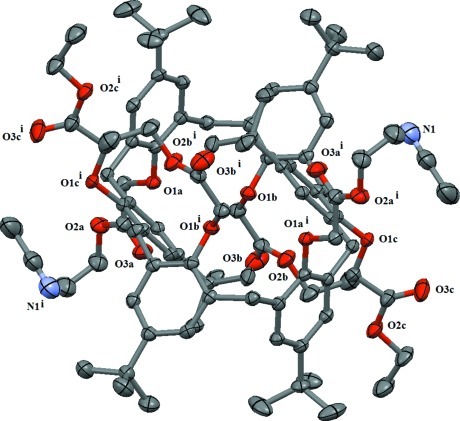
A view of the molecular structure of the title compound [only the hetero-atoms have been labelled; H atoms have been omitted for clarity; symmetry code: (i) -x+1, -y+2, -z].

**Fig. 3.**
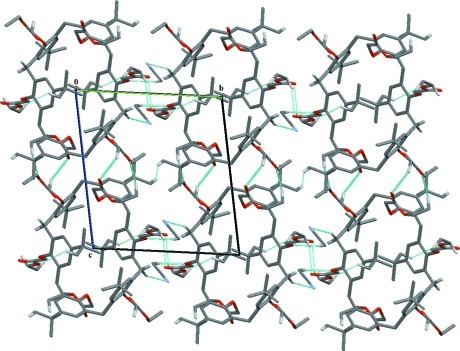
A view along the a axis of the crystal packing of the title compound. The C-H···O hydrogen bonds are shown as dashed cyan lines (see Table 1 for details; H atoms not involved in these interactions have been omitted for clarity).

## supplementary crystallographic information

### Comment

Calixarenes (Gutsche, 2008) have received considerable interest over the past four decades for their ability to entrap guest molecules. They have found applications in coordination (Homden & Redshaw, 2008; Gibson *et al.*, 1998) and supramolecular chemistry (Atwood *et al.*, 2002). They have also been investigated for their possible industrial applications such as, the catalysis of polymerization (Ling *et al.*, 2003), or metal ion receptors (Homden & Redshaw, 2008).

The title compound crystallizes as a diacetonotrile solvate, with half a molecule of calix[6]arene and one molecule of acetonitrile in the asymmetric unit (Fig. 1). The molecular structure of the title compound is illustrated in Fig. 2. The acetonitrile molecule is bound to the calix[6]arene *via* a weak C-H···O hydrogen bond involving an O atom of a lower rim substituent (Table 1). Due to the presence of the oxygen atoms, the lower rim substituents are suitable acceptors of weak C-H···O hydrogen bonds (intra- and intermolecular) from surrounding bridging methylene (C7) and methyl (C15) groups (Table 1). The shape of the calix[6]arene cavity is influenced by the presence of C—H···π interactions between the methyl group of the *tert*-butyl group and the aromatic ring C1c-C6c (Table 1 and Fig. 3).

The lower-rim substituent atoms (O3a,C14a,C15a) of ring C1a-C6a, are disordered over two position with an occupancy ratio of 0.897 (3) : 0.103 (3).

### Experimental

The title compound was prepared following a previously published procedure (McKervey *et al.* 1985).

### Refinement

Atoms (O3a,C14a,C15a), of the ethoxycarbonylmethoxy substituent on ring C1a-C6a, are disordered over two positions with an occupancy ratio of 0.897 (3) : 0.103 (3). Their positions were found from difference electron density maps. The disordered fragments were placed in appropriate positions, and all distances between neighbouring atoms were restrained, as well as the bond angles, to standard values. Site occupancies were refined for the different parts with the same thermal parameters for the same atoms in the various fragments. In the final cycles of refinement, the C-bound H-atoms were included in calculated positions and treated as riding atoms: C-H = 0.93, 0.97 and 0.96 Å for CH, CH_2_ and CH_3_ H-atoms, respectively, with U_iso_(H) = k × U_eq_(parent C-atom), where k = 1.5 for CH_3_ H-atoms and = 1.2 for other H-atoms.

### Crystal data

**Table:**

C_90_H_120_O_18_·2C_2_H_3_N	*Z* = 1
*M**_r_* = 1571.97	*F*(000) = 848
Triclinic, *P*1	*D*_x_ = 1.199 Mg m^−^^3^
Hall symbol: -P 1	Mo *K*α radiation, λ = 0.71073 Å
*a* = 12.6190 (2) Å	Cell parameters from 4823 reflections
*b* = 13.1500 (3) Å	θ = 1–22°
*c* = 14.8990 (4) Å	µ = 0.08 mm^−^^1^
α = 75.9037 (11)°	*T* = 150 K
β = 67.7646 (11)°	Prism, colourless
γ = 74.5815 (19)°	0.3 × 0.3 × 0.25 mm
*V* = 2177.67 (9) Å^3^	

### Data collection

**Table:**

Nonius KappaCCD area-detector diffractometer	7277 reflections with *I* > 2σ(*I*)
Radiation source: fine-focus sealed tube	*R*_int_ = 0.025
Graphite monochromator	θ_max_ = 27.5°, θ_min_ = 1.5°
Detector resolution: 9.091 pixels mm^-1^	*h* = −16→16
φ and ω scans	*k* = −17→17
19018 measured reflections	*l* = −19→19
9990 independent reflections	

### Refinement

**Table:**

Refinement on *F*^2^	Primary atom site location: structure-invariant direct methods
Least-squares matrix: full	Secondary atom site location: difference Fourier map
*R*[*F*^2^ > 2σ(*F*^2^)] = 0.061	Hydrogen site location: inferred from neighbouring sites
*wR*(*F*^2^) = 0.183	H-atom parameters constrained
*S* = 1.03	*w* = 1/[σ^2^(*F*_o_^2^) + (0.089*P*)^2^ + 1.397*P*] where *P* = (*F*_o_^2^ + 2*F*_c_^2^)/3
9990 reflections	(Δ/σ)_max_ < 0.001
538 parameters	Δρ_max_ = 0.43 e Å^−^^3^
12 restraints	Δρ_min_ = −0.32 e Å^−^^3^

### Special details

**Table:**

Geometry. All e.s.d.'s (except the e.s.d. in the dihedral angle between two l.s. planes) are estimated using the full covariance matrix. The cell e.s.d.'s are taken into account individually in the estimation of e.s.d.'s in distances, angles and torsion angles; correlations between e.s.d.'s in cell parameters are only used when they are defined by crystal symmetry. An approximate (isotropic) treatment of cell e.s.d.'s is used for estimating e.s.d.'s involving l.s. planes.
Refinement. Refinement of *F*^2^ against ALL reflections. The weighted *R*-factor *wR* and goodness of fit *S* are based on *F*^2^, conventional *R*-factors *R* are based on *F*, with *F* set to zero for negative *F*^2^. The threshold expression of *F*^2^ > σ(*F*^2^) is used only for calculating *R*-factors(gt) *etc*. and is not relevant to the choice of reflections for refinement. *R*-factors based on *F*^2^ are statistically about twice as large as those based on *F*, and *R*- factors based on ALL data will be even larger. The positions of disorder atoms were found from the electron density maps. Disodered fragments were then placed in appropriate positions, and all distances between neighbouring atoms were restrained as well as angles. Site occupancies were refined for the different parts with the same thermal parameters for the same atoms in various fragments. The final partial occupancies were found 0.896 (3). At the end of refinement, hydrogen atoms were placed in calculated positions with the thermal parameters *U*_iso_(H) (in the range 1.2–1.5 times *U*_eq_ of the parent atom)

### Fractional atomic coordinates and isotropic or equivalent isotropic displacement parameters (Å^2^)

**Table:**

	*x*	*y*	*z*	*U*_iso_*/*U*_eq_	Occ. (<1)
C1A	0.25078 (16)	1.17003 (15)	0.27628 (15)	0.0255 (4)	
C2A	0.21935 (16)	1.23763 (15)	0.19789 (14)	0.0253 (4)	
C3A	0.11776 (16)	1.22947 (16)	0.18578 (15)	0.0275 (4)	
H3A	0.0932	1.2775	0.1370	0.033*	
C4A	0.05112 (16)	1.15237 (16)	0.24363 (15)	0.0274 (4)	
C5A	0.08946 (16)	1.08385 (16)	0.31747 (15)	0.0274 (4)	
H5A	0.0483	1.0304	0.3559	0.033*	
C6A	0.18736 (16)	1.09210 (15)	0.33630 (15)	0.0265 (4)	
C7A	0.29444 (16)	1.31534 (15)	0.12584 (15)	0.0259 (4)	
H7A1	0.3264	1.3448	0.1615	0.031*	
H7A2	0.2453	1.3740	0.0976	0.031*	
C8A	−0.06086 (17)	1.14624 (18)	0.22848 (16)	0.0340 (5)	
C9A	−0.1624 (2)	1.2230 (3)	0.2855 (2)	0.0663 (9)	
H9A1	−0.1477	1.2944	0.2624	0.099*	
H9A2	−0.1714	1.2040	0.3541	0.099*	
H9A3	−0.2324	1.2192	0.2763	0.099*	
C10A	−0.0513 (2)	1.1786 (3)	0.1204 (2)	0.0524 (7)	
H10A	−0.1196	1.1685	0.1124	0.079*	
H10B	0.0166	1.1351	0.0815	0.079*	
H10C	−0.0447	1.2524	0.0994	0.079*	
C11A	−0.0862 (3)	1.0331 (2)	0.2613 (3)	0.0653 (9)	
H11A	−0.1008	1.0128	0.3306	0.098*	
H11B	−0.0203	0.9843	0.2268	0.098*	
H11C	−0.1536	1.0310	0.2469	0.098*	
C12A	0.32725 (17)	1.25402 (17)	0.35525 (16)	0.0316 (4)	
H12A	0.2754	1.2309	0.4202	0.038*	
H12B	0.2903	1.3237	0.3296	0.038*	
C13A	0.44232 (19)	1.26028 (18)	0.36074 (18)	0.0378 (5)	
O3A	0.42398 (15)	1.31975 (17)	0.42880 (16)	0.0397 (5)	0.897 (3)
C14A	0.5263 (2)	1.3287 (2)	0.4494 (2)	0.0450 (7)	0.897 (3)
H14A	0.5869	1.2658	0.4350	0.054*	0.897 (3)
H14B	0.5045	1.3328	0.5184	0.054*	0.897 (3)
C15A	0.5715 (3)	1.4255 (3)	0.3886 (3)	0.0607 (9)	0.897 (3)
H15A	0.6010	1.4178	0.3205	0.091*	0.897 (3)
H15B	0.6332	1.4342	0.4071	0.091*	0.897 (3)
H15C	0.5097	1.4870	0.3987	0.091*	0.897 (3)
O3E	0.4240 (13)	1.3615 (12)	0.3789 (15)	0.0397 (5)	0.103 (3)
C14E	0.5250 (18)	1.385 (2)	0.3852 (18)	0.0450 (7)	0.103 (3)
H14C	0.5585	1.4335	0.3267	0.054*	0.103 (3)
H14D	0.5826	1.3193	0.3865	0.054*	0.103 (3)
C15E	0.501 (3)	1.433 (2)	0.472 (2)	0.0607 (9)	0.103 (3)
H15D	0.4586	1.5048	0.4641	0.091*	0.103 (3)
H15E	0.5728	1.4324	0.4800	0.091*	0.103 (3)
H15F	0.4544	1.3918	0.5295	0.091*	0.103 (3)
O1A	0.34953 (11)	1.17950 (11)	0.29228 (10)	0.0280 (3)	
O2A	0.53644 (14)	1.21755 (15)	0.31260 (15)	0.0526 (5)	
C1B	0.37351 (16)	0.86213 (16)	0.34163 (14)	0.0264 (4)	
C2B	0.26097 (16)	0.90142 (15)	0.40260 (14)	0.0262 (4)	
C3B	0.18043 (17)	0.83401 (16)	0.44048 (15)	0.0281 (4)	
H3B	0.1068	0.8580	0.4834	0.034*	
C4B	0.20556 (17)	0.73175 (16)	0.41665 (15)	0.0289 (4)	
C5B	0.31505 (17)	0.69925 (16)	0.35082 (15)	0.0288 (4)	
H5B	0.3325	0.6329	0.3314	0.035*	
C6B	0.40056 (16)	0.76258 (16)	0.31235 (14)	0.0266 (4)	
C7B	0.22152 (17)	1.01623 (16)	0.42047 (15)	0.0287 (4)	
H7B1	0.2841	1.0385	0.4289	0.034*	
H7B2	0.1552	1.0207	0.4808	0.034*	
C8B	0.11157 (18)	0.66241 (17)	0.45974 (16)	0.0326 (5)	
C9B	0.0066 (2)	0.7205 (2)	0.4275 (2)	0.0498 (6)	
H9B1	−0.0240	0.7879	0.4508	0.075*	
H9B2	−0.0525	0.6778	0.4543	0.075*	
H9B3	0.0305	0.7324	0.3571	0.075*	
C10B	0.0715 (2)	0.6428 (2)	0.57235 (19)	0.0491 (6)	
H10D	0.1370	0.6072	0.5935	0.074*	
H10E	0.0133	0.5990	0.5980	0.074*	
H10F	0.0390	0.7100	0.5960	0.074*	
C11B	0.1572 (2)	0.5534 (2)	0.4241 (2)	0.0488 (6)	
H11D	0.1814	0.5642	0.3537	0.073*	
H11E	0.0963	0.5125	0.4514	0.073*	
H11F	0.2224	0.5154	0.4449	0.073*	
C12B	0.49621 (18)	0.95092 (18)	0.37165 (17)	0.0332 (5)	
H12C	0.4393	0.9374	0.4365	0.040*	
H12D	0.4963	1.0269	0.3564	0.040*	
C13B	0.61557 (18)	0.89186 (17)	0.37561 (16)	0.0327 (5)	
C14B	0.7921 (2)	0.7746 (2)	0.3031 (2)	0.0463 (6)	
H14E	0.8208	0.8064	0.3395	0.056*	
H14F	0.8450	0.7787	0.2358	0.056*	
C15B	0.7866 (2)	0.6599 (2)	0.3482 (2)	0.0542 (7)	
H15G	0.7424	0.6557	0.4170	0.081*	
H15H	0.8642	0.6193	0.3397	0.081*	
H15I	0.7497	0.6314	0.3167	0.081*	
O1B	0.45860 (12)	0.92449 (11)	0.30288 (10)	0.0297 (3)	
O2B	0.65196 (15)	0.90149 (16)	0.43608 (14)	0.0545 (5)	
O3B	0.67560 (13)	0.83237 (13)	0.30542 (13)	0.0426 (4)	
C1C	0.60359 (16)	0.70774 (14)	0.05452 (15)	0.0247 (4)	
C2C	0.51242 (16)	0.75340 (15)	0.13118 (15)	0.0256 (4)	
C3C	0.42030 (16)	0.82565 (15)	0.10770 (15)	0.0269 (4)	
H3C	0.3587	0.8569	0.1575	0.032*	
C4C	0.41697 (16)	0.85299 (15)	0.01170 (15)	0.0253 (4)	
C5C	0.51041 (16)	0.80732 (15)	−0.06241 (15)	0.0256 (4)	
H5C	0.5100	0.8259	−0.1266	0.031*	
C6C	0.60533 (15)	0.73392 (14)	−0.04272 (14)	0.0243 (4)	
C7C	0.51460 (17)	0.72522 (17)	0.23651 (15)	0.0299 (4)	
H7C1	0.5366	0.6482	0.2523	0.036*	
H7C2	0.5741	0.7566	0.2399	0.036*	
C8C	0.31113 (16)	0.93202 (16)	−0.00769 (15)	0.0282 (4)	
C9C	0.20150 (19)	0.8836 (2)	0.0495 (2)	0.0426 (6)	
H9C1	0.2115	0.8169	0.0290	0.064*	
H9C2	0.1351	0.9319	0.0368	0.064*	
H9C3	0.1894	0.8716	0.1186	0.064*	
C10C	0.2975 (2)	1.03574 (18)	0.0273 (2)	0.0441 (6)	
H10G	0.2860	1.0216	0.0964	0.066*	
H10H	0.2314	1.0857	0.0156	0.066*	
H10I	0.3666	1.0653	−0.0079	0.066*	
C11C	0.3238 (2)	0.9565 (2)	−0.11688 (18)	0.0444 (6)	
H11G	0.3915	0.9876	−0.1539	0.067*	
H11H	0.2556	1.0057	−0.1256	0.067*	
H11I	0.3320	0.8915	−0.1395	0.067*	
C12C	0.78085 (16)	0.67061 (16)	0.08537 (16)	0.0298 (4)	
H12E	0.7474	0.7119	0.1388	0.036*	
H12F	0.8133	0.7175	0.0251	0.036*	
C13C	0.87500 (19)	0.58009 (18)	0.10460 (17)	0.0360 (5)	
C14C	1.0527 (2)	0.5423 (2)	0.1371 (2)	0.0515 (7)	
H14G	1.0990	0.5093	0.0792	0.062*	
H14H	1.0270	0.4868	0.1919	0.062*	
C15C	1.1226 (2)	0.6006 (3)	0.1588 (3)	0.0650 (9)	
H15J	1.1469	0.6558	0.1045	0.097*	
H15K	1.1901	0.5519	0.1694	0.097*	
H15L	1.0764	0.6320	0.2168	0.097*	
O1C	0.69366 (11)	0.62850 (10)	0.07719 (10)	0.0277 (3)	
O2C	0.95249 (13)	0.61902 (12)	0.12039 (13)	0.0392 (4)	
O3C	0.87997 (18)	0.48727 (14)	0.10612 (19)	0.0659 (6)	
N1	0.4238 (3)	0.4887 (2)	0.1912 (2)	0.0724 (8)	
C2	0.5136 (3)	0.4516 (2)	0.1443 (3)	0.0590 (7)	
C3	0.6277 (3)	0.4032 (3)	0.0847 (3)	0.0849 (12)	
H3D	0.6479	0.3308	0.1148	0.127*	
H3E	0.6842	0.4428	0.0793	0.127*	
H3F	0.6267	0.4037	0.0205	0.127*	

### Atomic displacement parameters (Å^2^)

**Table:**

	*U*^11^	*U*^22^	*U*^33^	*U*^12^	*U*^13^	*U*^23^
C1A	0.0210 (9)	0.0266 (10)	0.0316 (10)	−0.0015 (7)	−0.0105 (8)	−0.0097 (8)
C2A	0.0220 (9)	0.0232 (9)	0.0306 (10)	0.0004 (7)	−0.0091 (8)	−0.0088 (8)
C3A	0.0251 (9)	0.0286 (10)	0.0287 (10)	0.0007 (7)	−0.0120 (8)	−0.0059 (8)
C4A	0.0200 (9)	0.0319 (10)	0.0311 (10)	−0.0008 (7)	−0.0091 (8)	−0.0102 (8)
C5A	0.0237 (9)	0.0283 (10)	0.0304 (10)	−0.0059 (7)	−0.0105 (8)	−0.0019 (8)
C6A	0.0244 (9)	0.0266 (10)	0.0295 (10)	−0.0009 (7)	−0.0106 (8)	−0.0080 (8)
C7A	0.0246 (9)	0.0220 (9)	0.0311 (10)	−0.0013 (7)	−0.0114 (8)	−0.0045 (8)
C8A	0.0242 (9)	0.0440 (12)	0.0383 (12)	−0.0073 (9)	−0.0145 (9)	−0.0072 (10)
C9A	0.0277 (12)	0.106 (3)	0.075 (2)	0.0032 (14)	−0.0202 (13)	−0.0463 (19)
C10A	0.0420 (13)	0.081 (2)	0.0473 (15)	−0.0239 (13)	−0.0255 (12)	−0.0032 (13)
C11A	0.0664 (18)	0.0657 (19)	0.087 (2)	−0.0374 (15)	−0.0521 (17)	0.0155 (16)
C12A	0.0291 (10)	0.0324 (11)	0.0374 (11)	−0.0040 (8)	−0.0124 (9)	−0.0133 (9)
C13A	0.0332 (11)	0.0364 (12)	0.0483 (14)	−0.0067 (9)	−0.0144 (10)	−0.0134 (10)
O3A	0.0367 (9)	0.0467 (12)	0.0478 (12)	−0.0112 (8)	−0.0189 (9)	−0.0178 (10)
C14A	0.0480 (15)	0.0444 (15)	0.0549 (18)	−0.0106 (12)	−0.0273 (14)	−0.0115 (13)
C15A	0.0574 (19)	0.0566 (19)	0.080 (2)	−0.0189 (16)	−0.0309 (17)	−0.0095 (17)
O3E	0.0367 (9)	0.0467 (12)	0.0478 (12)	−0.0112 (8)	−0.0189 (9)	−0.0178 (10)
C14E	0.0480 (15)	0.0444 (15)	0.0549 (18)	−0.0106 (12)	−0.0273 (14)	−0.0115 (13)
C15E	0.0574 (19)	0.0566 (19)	0.080 (2)	−0.0189 (16)	−0.0309 (17)	−0.0095 (17)
O1A	0.0241 (6)	0.0296 (7)	0.0350 (8)	−0.0031 (5)	−0.0138 (6)	−0.0097 (6)
O2A	0.0294 (8)	0.0567 (11)	0.0805 (14)	−0.0052 (8)	−0.0154 (8)	−0.0348 (10)
C1B	0.0228 (9)	0.0307 (10)	0.0249 (10)	−0.0057 (7)	−0.0105 (7)	0.0016 (8)
C2B	0.0270 (9)	0.0284 (10)	0.0248 (10)	−0.0020 (7)	−0.0128 (8)	−0.0041 (8)
C3B	0.0237 (9)	0.0322 (10)	0.0269 (10)	−0.0034 (8)	−0.0073 (8)	−0.0062 (8)
C4B	0.0278 (10)	0.0309 (10)	0.0296 (10)	−0.0062 (8)	−0.0124 (8)	−0.0025 (8)
C5B	0.0303 (10)	0.0263 (10)	0.0320 (11)	−0.0025 (8)	−0.0141 (8)	−0.0059 (8)
C6B	0.0253 (9)	0.0303 (10)	0.0249 (10)	−0.0017 (8)	−0.0131 (8)	−0.0023 (8)
C7B	0.0268 (9)	0.0293 (10)	0.0306 (10)	−0.0041 (8)	−0.0112 (8)	−0.0049 (8)
C8B	0.0309 (10)	0.0308 (11)	0.0382 (12)	−0.0092 (8)	−0.0109 (9)	−0.0063 (9)
C9B	0.0386 (13)	0.0531 (15)	0.0652 (17)	−0.0135 (11)	−0.0218 (12)	−0.0107 (13)
C10B	0.0524 (15)	0.0524 (15)	0.0450 (14)	−0.0246 (12)	−0.0117 (12)	−0.0038 (12)
C11B	0.0434 (13)	0.0414 (13)	0.0622 (17)	−0.0149 (11)	−0.0113 (12)	−0.0126 (12)
C12B	0.0305 (10)	0.0350 (11)	0.0389 (12)	−0.0049 (8)	−0.0163 (9)	−0.0083 (9)
C13B	0.0321 (10)	0.0320 (11)	0.0375 (12)	−0.0078 (8)	−0.0150 (9)	−0.0051 (9)
C14B	0.0313 (11)	0.0516 (15)	0.0641 (17)	0.0044 (10)	−0.0266 (11)	−0.0201 (13)
C15B	0.0471 (14)	0.0595 (17)	0.0514 (16)	0.0018 (12)	−0.0214 (12)	−0.0060 (13)
O1B	0.0259 (7)	0.0327 (7)	0.0330 (8)	−0.0084 (6)	−0.0114 (6)	−0.0041 (6)
O2B	0.0456 (10)	0.0742 (13)	0.0574 (11)	0.0042 (9)	−0.0312 (9)	−0.0295 (10)
O3B	0.0321 (8)	0.0462 (9)	0.0571 (11)	0.0069 (7)	−0.0249 (8)	−0.0215 (8)
C1C	0.0214 (8)	0.0183 (9)	0.0337 (10)	−0.0028 (7)	−0.0110 (8)	−0.0012 (7)
C2C	0.0231 (9)	0.0235 (9)	0.0297 (10)	−0.0044 (7)	−0.0096 (8)	−0.0023 (8)
C3C	0.0216 (9)	0.0260 (10)	0.0301 (10)	−0.0027 (7)	−0.0065 (8)	−0.0047 (8)
C4C	0.0213 (8)	0.0218 (9)	0.0330 (10)	−0.0037 (7)	−0.0114 (8)	−0.0013 (8)
C5C	0.0242 (9)	0.0246 (9)	0.0293 (10)	−0.0055 (7)	−0.0111 (8)	−0.0025 (8)
C6C	0.0209 (8)	0.0203 (9)	0.0324 (10)	−0.0056 (7)	−0.0090 (7)	−0.0036 (8)
C7C	0.0250 (9)	0.0328 (11)	0.0310 (11)	−0.0003 (8)	−0.0131 (8)	−0.0034 (8)
C8C	0.0208 (9)	0.0271 (10)	0.0344 (11)	−0.0009 (7)	−0.0107 (8)	−0.0027 (8)
C9C	0.0269 (10)	0.0430 (13)	0.0560 (15)	−0.0083 (9)	−0.0160 (10)	0.0004 (11)
C10C	0.0409 (12)	0.0277 (11)	0.0687 (17)	0.0028 (9)	−0.0292 (12)	−0.0093 (11)
C11C	0.0340 (11)	0.0512 (14)	0.0427 (13)	0.0061 (10)	−0.0201 (10)	−0.0018 (11)
C12C	0.0239 (9)	0.0281 (10)	0.0352 (11)	−0.0013 (8)	−0.0104 (8)	−0.0042 (8)
C13C	0.0333 (11)	0.0333 (12)	0.0423 (13)	0.0039 (9)	−0.0186 (10)	−0.0094 (9)
C14C	0.0406 (13)	0.0481 (15)	0.0752 (19)	0.0128 (11)	−0.0369 (13)	−0.0217 (13)
C15C	0.0427 (15)	0.0656 (19)	0.100 (3)	0.0083 (13)	−0.0380 (16)	−0.0343 (18)
O1C	0.0229 (6)	0.0230 (7)	0.0355 (8)	0.0005 (5)	−0.0131 (6)	−0.0019 (6)
O2C	0.0311 (8)	0.0374 (8)	0.0541 (10)	0.0072 (6)	−0.0238 (7)	−0.0158 (7)
O3C	0.0627 (12)	0.0335 (10)	0.1216 (19)	0.0093 (8)	−0.0620 (13)	−0.0182 (11)
N1	0.0620 (17)	0.0630 (16)	0.097 (2)	−0.0198 (13)	−0.0187 (16)	−0.0259 (16)
C2	0.0565 (17)	0.0495 (16)	0.077 (2)	−0.0222 (14)	−0.0171 (16)	−0.0163 (15)
C3	0.068 (2)	0.070 (2)	0.111 (3)	−0.0290 (18)	0.003 (2)	−0.042 (2)

### Geometric parameters (Å, º)

**Table:**

C1A—C6A	1.392 (3)	C9B—H9B2	0.9600
C1A—O1A	1.395 (2)	C9B—H9B3	0.9600
C1A—C2A	1.403 (3)	C10B—H10D	0.9600
C2A—C3A	1.395 (3)	C10B—H10E	0.9600
C2A—C7A	1.518 (3)	C10B—H10F	0.9600
C3A—C4A	1.399 (3)	C11B—H11D	0.9600
C3A—H3A	0.9300	C11B—H11E	0.9600
C4A—C5A	1.394 (3)	C11B—H11F	0.9600
C4A—C8A	1.538 (3)	C12B—O1B	1.423 (2)
C5A—C6A	1.402 (3)	C12B—C13B	1.516 (3)
C5A—H5A	0.9300	C12B—H12C	0.9700
C6A—C7B	1.525 (3)	C12B—H12D	0.9700
C7A—C6C^i^	1.524 (3)	C13B—O2B	1.199 (3)
C7A—H7A1	0.9700	C13B—O3B	1.328 (3)
C7A—H7A2	0.9700	C14B—O3B	1.456 (3)
C8A—C9A	1.512 (3)	C14B—C15B	1.504 (4)
C8A—C10A	1.528 (3)	C14B—H14E	0.9700
C8A—C11A	1.529 (4)	C14B—H14F	0.9700
C9A—H9A1	0.9600	C15B—H15G	0.9600
C9A—H9A2	0.9600	C15B—H15H	0.9600
C9A—H9A3	0.9600	C15B—H15I	0.9600
C10A—H10A	0.9600	C1C—C6C	1.398 (3)
C10A—H10B	0.9600	C1C—C2C	1.400 (3)
C10A—H10C	0.9600	C1C—O1C	1.408 (2)
C11A—H11A	0.9600	C2C—C3C	1.391 (3)
C11A—H11B	0.9600	C2C—C7C	1.531 (3)
C11A—H11C	0.9600	C3C—C4C	1.401 (3)
C12A—O1A	1.422 (2)	C3C—H3C	0.9300
C12A—C13A	1.510 (3)	C4C—C5C	1.390 (3)
C12A—H12A	0.9700	C4C—C8C	1.535 (3)
C12A—H12B	0.9700	C5C—C6C	1.402 (3)
C13A—O2A	1.199 (3)	C5C—H5C	0.9300
C13A—O3A	1.344 (3)	C6C—C7A^i^	1.524 (3)
C13A—O3E	1.365 (13)	C7C—H7C1	0.9700
O3A—C14A	1.474 (3)	C7C—H7C2	0.9700
C14A—C15A	1.483 (4)	C8C—C10C	1.522 (3)
C14A—H14A	0.9700	C8C—C11C	1.533 (3)
C14A—H14B	0.9700	C8C—C9C	1.536 (3)
C15A—H15A	0.9600	C9C—H9C1	0.9600
C15A—H15B	0.9600	C9C—H9C2	0.9600
C15A—H15C	0.9600	C9C—H9C3	0.9600
O3E—C14E	1.428 (17)	C10C—H10G	0.9600
C14E—C15E	1.471 (18)	C10C—H10H	0.9600
C14E—H14C	0.9700	C10C—H10I	0.9600
C14E—H14D	0.9700	C11C—H11G	0.9600
C15E—H15D	0.9600	C11C—H11H	0.9600
C15E—H15E	0.9600	C11C—H11I	0.9600
C15E—H15F	0.9600	C12C—O1C	1.411 (2)
C1B—O1B	1.388 (2)	C12C—C13C	1.500 (3)
C1B—C6B	1.396 (3)	C12C—H12E	0.9700
C1B—C2B	1.404 (3)	C12C—H12F	0.9700
C2B—C3B	1.392 (3)	C13C—O3C	1.201 (3)
C2B—C7B	1.515 (3)	C13C—O2C	1.331 (3)
C3B—C4B	1.398 (3)	C14C—O2C	1.455 (3)
C3B—H3B	0.9300	C14C—C15C	1.471 (4)
C4B—C5B	1.385 (3)	C14C—H14G	0.9700
C4B—C8B	1.538 (3)	C14C—H14H	0.9700
C5B—C6B	1.401 (3)	C15C—H15J	0.9600
C5B—H5B	0.9300	C15C—H15K	0.9600
C6B—C7C	1.501 (3)	C15C—H15L	0.9600
C7B—H7B1	0.9700	N1—C2	1.133 (4)
C7B—H7B2	0.9700	C2—C3	1.444 (5)
C8B—C9B	1.528 (3)	C3—H3D	0.9600
C8B—C10B	1.534 (3)	C3—H3E	0.9600
C8B—C11B	1.540 (3)	C3—H3F	0.9600
C9B—H9B1	0.9600		
			
C6A—C1A—O1A	119.63 (17)	H9B1—C9B—H9B3	109.5
C6A—C1A—C2A	121.28 (17)	H9B2—C9B—H9B3	109.5
O1A—C1A—C2A	119.06 (17)	C8B—C10B—H10D	109.5
C3A—C2A—C1A	117.95 (18)	C8B—C10B—H10E	109.5
C3A—C2A—C7A	120.25 (18)	H10D—C10B—H10E	109.5
C1A—C2A—C7A	121.77 (16)	C8B—C10B—H10F	109.5
C2A—C3A—C4A	122.99 (18)	H10D—C10B—H10F	109.5
C2A—C3A—H3A	118.5	H10E—C10B—H10F	109.5
C4A—C3A—H3A	118.5	C8B—C11B—H11D	109.5
C5A—C4A—C3A	116.58 (17)	C8B—C11B—H11E	109.5
C5A—C4A—C8A	121.89 (18)	H11D—C11B—H11E	109.5
C3A—C4A—C8A	121.50 (18)	C8B—C11B—H11F	109.5
C4A—C5A—C6A	122.83 (18)	H11D—C11B—H11F	109.5
C4A—C5A—H5A	118.6	H11E—C11B—H11F	109.5
C6A—C5A—H5A	118.6	O1B—C12B—C13B	116.20 (18)
C1A—C6A—C5A	118.19 (18)	O1B—C12B—H12C	108.2
C1A—C6A—C7B	122.14 (17)	C13B—C12B—H12C	108.2
C5A—C6A—C7B	119.67 (17)	O1B—C12B—H12D	108.2
C2A—C7A—C6C^i^	114.23 (15)	C13B—C12B—H12D	108.2
C2A—C7A—H7A1	108.7	H12C—C12B—H12D	107.4
C6C^i^—C7A—H7A1	108.7	O2B—C13B—O3B	124.0 (2)
C2A—C7A—H7A2	108.7	O2B—C13B—C12B	121.8 (2)
C6C^i^—C7A—H7A2	108.7	O3B—C13B—C12B	114.18 (18)
H7A1—C7A—H7A2	107.6	O3B—C14B—C15B	108.9 (2)
C9A—C8A—C10A	107.5 (2)	O3B—C14B—H14E	109.9
C9A—C8A—C11A	109.8 (2)	C15B—C14B—H14E	109.9
C10A—C8A—C11A	107.2 (2)	O3B—C14B—H14F	109.9
C9A—C8A—C4A	109.27 (18)	C15B—C14B—H14F	109.9
C10A—C8A—C4A	111.33 (18)	H14E—C14B—H14F	108.3
C11A—C8A—C4A	111.63 (19)	C14B—C15B—H15G	109.5
C8A—C9A—H9A1	109.5	C14B—C15B—H15H	109.5
C8A—C9A—H9A2	109.5	H15G—C15B—H15H	109.5
H9A1—C9A—H9A2	109.5	C14B—C15B—H15I	109.5
C8A—C9A—H9A3	109.5	H15G—C15B—H15I	109.5
H9A1—C9A—H9A3	109.5	H15H—C15B—H15I	109.5
H9A2—C9A—H9A3	109.5	C1B—O1B—C12B	116.27 (16)
C8A—C10A—H10A	109.5	C13B—O3B—C14B	117.53 (18)
C8A—C10A—H10B	109.5	C6C—C1C—C2C	122.47 (17)
H10A—C10A—H10B	109.5	C6C—C1C—O1C	118.85 (17)
C8A—C10A—H10C	109.5	C2C—C1C—O1C	118.60 (17)
H10A—C10A—H10C	109.5	C3C—C2C—C1C	117.35 (18)
H10B—C10A—H10C	109.5	C3C—C2C—C7C	121.44 (17)
C8A—C11A—H11A	109.5	C1C—C2C—C7C	121.21 (17)
C8A—C11A—H11B	109.5	C2C—C3C—C4C	122.32 (18)
H11A—C11A—H11B	109.5	C2C—C3C—H3C	118.8
C8A—C11A—H11C	109.5	C4C—C3C—H3C	118.8
H11A—C11A—H11C	109.5	C5C—C4C—C3C	118.37 (17)
H11B—C11A—H11C	109.5	C5C—C4C—C8C	122.59 (18)
O1A—C12A—C13A	108.09 (16)	C3C—C4C—C8C	119.04 (17)
O1A—C12A—H12A	110.1	C4C—C5C—C6C	121.61 (18)
C13A—C12A—H12A	110.1	C4C—C5C—H5C	119.2
O1A—C12A—H12B	110.1	C6C—C5C—H5C	119.2
C13A—C12A—H12B	110.1	C1C—C6C—C5C	117.86 (17)
H12A—C12A—H12B	108.4	C1C—C6C—C7A^i^	122.08 (16)
O2A—C13A—O3A	124.7 (2)	C5C—C6C—C7A^i^	120.06 (17)
O2A—C13A—O3E	121.1 (7)	C6B—C7C—C2C	114.14 (16)
O3A—C13A—O3E	34.8 (8)	C6B—C7C—H7C1	108.7
O2A—C13A—C12A	125.3 (2)	C2C—C7C—H7C1	108.7
O3A—C13A—C12A	110.00 (18)	C6B—C7C—H7C2	108.7
O3E—C13A—C12A	103.4 (6)	C2C—C7C—H7C2	108.7
C13A—O3A—C14A	117.7 (2)	H7C1—C7C—H7C2	107.6
O3A—C14A—C15A	110.2 (2)	C10C—C8C—C11C	108.91 (19)
O3A—C14A—H14A	109.6	C10C—C8C—C4C	108.87 (16)
C15A—C14A—H14A	109.6	C11C—C8C—C4C	112.28 (17)
O3A—C14A—H14B	109.6	C10C—C8C—C9C	109.64 (19)
C15A—C14A—H14B	109.6	C11C—C8C—C9C	108.20 (18)
H14A—C14A—H14B	108.1	C4C—C8C—C9C	108.92 (17)
C13A—O3E—C14E	112.4 (14)	C8C—C9C—H9C1	109.5
O3E—C14E—C15E	113.4 (18)	C8C—C9C—H9C2	109.5
O3E—C14E—H14C	108.9	H9C1—C9C—H9C2	109.5
C15E—C14E—H14C	108.9	C8C—C9C—H9C3	109.5
O3E—C14E—H14D	108.9	H9C1—C9C—H9C3	109.5
C15E—C14E—H14D	108.9	H9C2—C9C—H9C3	109.5
H14C—C14E—H14D	107.7	C8C—C10C—H10G	109.5
C14E—C15E—H15D	109.5	C8C—C10C—H10H	109.5
C14E—C15E—H15E	109.5	H10G—C10C—H10H	109.5
H15D—C15E—H15E	109.5	C8C—C10C—H10I	109.5
C14E—C15E—H15F	109.5	H10G—C10C—H10I	109.5
H15D—C15E—H15F	109.5	H10H—C10C—H10I	109.5
H15E—C15E—H15F	109.5	C8C—C11C—H11G	109.5
C1A—O1A—C12A	114.19 (14)	C8C—C11C—H11H	109.5
O1B—C1B—C6B	117.99 (17)	H11G—C11C—H11H	109.5
O1B—C1B—C2B	120.86 (18)	C8C—C11C—H11I	109.5
C6B—C1B—C2B	120.99 (17)	H11G—C11C—H11I	109.5
C3B—C2B—C1B	117.93 (18)	H11H—C11C—H11I	109.5
C3B—C2B—C7B	119.28 (17)	O1C—C12C—C13C	108.95 (16)
C1B—C2B—C7B	122.54 (18)	O1C—C12C—H12E	109.9
C2B—C3B—C4B	122.77 (18)	C13C—C12C—H12E	109.9
C2B—C3B—H3B	118.6	O1C—C12C—H12F	109.9
C4B—C3B—H3B	118.6	C13C—C12C—H12F	109.9
C5B—C4B—C3B	117.22 (18)	H12E—C12C—H12F	108.3
C5B—C4B—C8B	122.75 (18)	O3C—C13C—O2C	125.2 (2)
C3B—C4B—C8B	119.98 (18)	O3C—C13C—C12C	125.8 (2)
C4B—C5B—C6B	122.44 (19)	O2C—C13C—C12C	109.03 (18)
C4B—C5B—H5B	118.8	O2C—C14C—C15C	107.7 (2)
C6B—C5B—H5B	118.8	O2C—C14C—H14G	110.2
C1B—C6B—C5B	118.37 (18)	C15C—C14C—H14G	110.2
C1B—C6B—C7C	122.02 (18)	O2C—C14C—H14H	110.2
C5B—C6B—C7C	119.48 (18)	C15C—C14C—H14H	110.2
C2B—C7B—C6A	112.35 (16)	H14G—C14C—H14H	108.5
C2B—C7B—H7B1	109.1	C14C—C15C—H15J	109.5
C6A—C7B—H7B1	109.1	C14C—C15C—H15K	109.5
C2B—C7B—H7B2	109.1	H15J—C15C—H15K	109.5
C6A—C7B—H7B2	109.1	C14C—C15C—H15L	109.5
H7B1—C7B—H7B2	107.9	H15J—C15C—H15L	109.5
C9B—C8B—C10B	108.7 (2)	H15K—C15C—H15L	109.5
C9B—C8B—C4B	109.02 (18)	C1C—O1C—C12C	113.28 (14)
C10B—C8B—C4B	110.53 (18)	C13C—O2C—C14C	116.60 (18)
C9B—C8B—C11B	108.6 (2)	N1—C2—C3	179.4 (3)
C10B—C8B—C11B	108.4 (2)	C2—C3—H3D	109.5
C4B—C8B—C11B	111.50 (18)	C2—C3—H3E	109.5
C8B—C9B—H9B1	109.5	H3D—C3—H3E	109.5
C8B—C9B—H9B2	109.5	C2—C3—H3F	109.5
H9B1—C9B—H9B2	109.5	H3D—C3—H3F	109.5
C8B—C9B—H9B3	109.5	H3E—C3—H3F	109.5

Symmetry code: (i) −*x*+1, −*y*+2, −*z*.

### Hydrogen-bond geometry (Å, º)

Cg1 is the centroid of the C1C–C6C ring.

**Table:**

*D*—H···*A*	*D*—H	H···*A*	*D*···*A*	*D*—H···*A*
C3—H3*E*···O1*C*	0.96	2.47	3.250 (4)	139
C7*C*—H7*C*2···O3*B*	0.97	2.38	3.312 (3)	160
C12*B*—H12*D*···O2*A*	0.97	2.57	3.526 (3)	169
C15*B*—H15*G*···O3*A*^ii^	0.96	2.48	3.398	160
C5*C*—H5*C*···O1*A*^i^	0.93	2.44	3.190 (2)	138
C11*C*—H11*G*···O1*B*^i^	0.96	2.56	3.477 (3)	159
C10*C*—H10*I*···*Cg*1^i^	0.96	2.73	3.588 (3)	148

Symmetry codes: (i) −*x*+1, −*y*+2, −*z*; (ii) −*x*+1, −*y*+2, −*z*+1.
